# Identification of EIL and ERF Genes Related to Fruit Ripening in Peach

**DOI:** 10.3390/ijms21082846

**Published:** 2020-04-19

**Authors:** Hui Zhou, Lei Zhao, Qiurui Yang, Mohamed Hamdy Amar, Collins Ogutu, Qian Peng, Liao Liao, Jinyun Zhang, Yuepeng Han

**Affiliations:** 1 CAS Key Laboratory of Plant Germplasm Enhancement and Specialty Agriculture, Wuhan Botanical Garden, The Innovative Academy of Seed Design, Chinese Academy of Sciences, Wuhan 430074, China; zhouhui@aaas.org.cn (H.Z.); zhaolei16@mails.ucas.ac.cn (L.Z.); yangqiurui16@mails.ucas.ac.cn (Q.Y.); mohamed.amar@wbgcas.cn (M.H.A.); collins@wbgcas.cn (C.O.); pengqian114@mails.ucas.ac.cn (Q.P.); liao168000@wbgcas.cn (L.L.); 2 Key Laboratory of Genetic Improvement and Ecophysiology of Horticultural Crops, Institute of Horticulture, Anhui Academy of Agricultural Sciences, Hefei 230031, China; zjy600@aaas.org.cn; 3 University of Chinese Academy of Sciences, 19A Yuquanlu, Beijing 100049, China; 4 Sino-African Joint Research Center, Chinese Academy of Sciences, Wuhan 430074, China; 5 Center of Economic Botany, Core Botanical Gardens, Chinese Academy of Sciences, Wuhan 430074, China

**Keywords:** *Prunus persica*, ethylene, ERF, EIL, fruit ripening

## Abstract

Peach (*Prunus persica*) is a climacteric fruit with a relatively short shelf life due to its fast ripening or softening process. Here, we report the association of gene families encoding ethylene insensitive-3 like (EIL) and ethylene response factor (ERF) with fruit ripening in peach. In total, 3 *PpEILs* and 12 PpERFs were highly expressed in fruit, with the majority showing a peak of expression at different stages. All three EILs could activate ethylene biosynthesis genes *PpACS1* and *PpACO1*. One out of the 12 *PpERFs*, termed *PpERF.E2*, is a homolog of ripening-associated *ERFs* in tomato, with a consistently high expression throughout fruit development and an ability to activate *PpACS1* and *PpACO1*. Additionally, four subgroup F PpERFs harboring the EAR repressive motif were able to repress the *PpACO1* promoter but could also activate the *PpACS1* promoter. Promoter deletion assay revealed that PpEILs and PpERFs could participate in transcriptional regulation of *PpACS1* through either direct or indirect interaction with various cis-elements. Taken together, these results suggested that all three *PpEILs* and *PpERF.E2* are candidates involved in ethylene biosynthesis, and EAR motif-containing *PpERFs* may function as activator or repressor of ethylene biosynthesis genes in peach. Our study provides an insight into the roles of EILs and ERFs in the fruit ripening process.

## 1. Introduction

Fruit ripening is a coordinated developmental process that leads to dramatic changes in the texture, color, flavor, and aroma of the fruit flesh. Ethylene plays a pivotal regulatory role in the fruit ripening process of most fleshy fruits [1,2,3]. Based on the intervention of ethylene during ripening, fruits can be divided into two categories: Climacteric fruits with a rapid rise in respiration and a burst of ethylene biosynthesis at the onset of ripening and non-climacteric fruits with no dramatic increase in respiration and ethylene biosynthesis. In climacteric fruits, the basal rate of ethylene production during development is often defined as system 1 ethylene, whilst system 2 refers to the high rate of autocatalytic ethylene synthesis during ripening [4].

The ethylene biosynthesis in plants starts with the precursor *S*-adenosylmethionine (*S*-AdoMet), which is synthesized from the methionine by *S*-AdoMet synthetase [5]. The *S*-AdoMet is converted to 1-aminocyclopropane-1-carboxylic acid (ACC) by ACC synthase (ACS), and subsequently oxidized to ethylene via ACC oxidase (ACO). Both ACS and ACO are rate-limiting enzymes and encoded by multigene families [6,7,8,9]. Transcription factors (TFs) encoding ethylene-insensitive 3 (EIN3) and EIN3-like (EIL) proteins play important roles in the ethylene signaling of higher plants [5,7]. The *EIN3* gene activates transcription of ethylene response genes, such as *ERF1*, through binding directly to a primary ethylene response element (PERE) [10,11], whereas, the *EIL* gene can bind to various PERE-like elements [12,13]. The transcriptional activity of *EILs* is regulated by phosphorylation within the EIN3/EIL phosphorylation region (EPR), which promotes the dimerization process to initiate the *EIL*-mediated transcription of early ethylene-regulated genes [14,15]. The *EIL* genes show an expression divergence among species, with a peak level during fruit development [16,17,18] or ripening [19,20,21]. In addition, *EILs* may promote ethylene production by activating transcription of genes encoding rate-limiting enzymes in ethylene biosynthesis [7,18]. 

ERF TFs belong to the APETALA2 (AP2)/ERF superfamily, which is characterized by a conserved AP2/ERF DNA binding domain, comprising of 60–70 amino acid residues [22]. There are 122 and 77 ERF TFs in the *Arabidopsis* and tomato genomes, respectively [22,23]. ERF TFs are involved in various biological processes, such as development [24,25], metabolism [26,27,28], plant hormone signaling [29,30,31,32], stress resistance [33,34,35], and fruit ripening [27,36,37]. ERF TFs regulate fruit ripening by directly binding to the GCC-box with a core AGCCGCC sequence or dehydration-responsive element (DRE) motif of their downstream genes, such as *ACS* and *ACO3* of tomato [38,39]. However, both the GCC-box and DRE motif are absent in the promoter of ERF target genes, such as *ACO54* in pear [40] and *XET5* in kiwifruit [18], which suggests the possibility of other unknown *cis*-element(s) recognized by ERF TFs. 

ERF TFs are divided into positive and negative regulators based on the activation or repression function of conserved motifs outside of the AP2/ERF domain [22]. Transcriptional activation motifs are probably related to acidic regions but show no sequence conservation [41]. By contrast, the transcriptional repression function is usually usually due to the ERF-associated amphiphilic repression (EAR) motif (LxLxL or DLNxxP) in the C-terminal region [18,42,43]. EAR motif-mediated repression probably occurs through chromatin modification of regulatory regions by histone deacetylation via physically interaction with co-repressors, such as Sin3-associated polypeptide of 18 kDa (SAP18) or TOPLESS (TPL) [44,45]. 

Peach (*Prunus persica*) is a typical climacteric fruit with a short shelf life. PpACS1 (Prupe.2G176900) and PpACO1 (Prupe.3G209900) are the rate-limiting enzymes for autocatalytic ethylene synthesis during the ripening stage in peach [46,47,48]. In addition, isolation of *EIL* and *ERF* genes and their expression profiles have been reported in peach but with no functional assessment [49,50,51]. In this study, functional analysis was conducted for 3 members of the *EIL* family and 12 members of the *ERF* family using the dual luciferase assay. All three *EIL* genes, *PpEIL1*-*3*, and an *ERF* gene *PpERF.E2* were found to be candidates involved in fruit ripening. Interestingly, the repressor-type *ERF* genes showed the ability to activate transcription of the ethylene biosynthetic gene *PpACS1*. Our results provide an insight into ethylene signal transduction in the fruits of peach. 

## 2. Results

### 2.1. Identification of EIL and ERF Genes That Are Expressed in Peach Fruit

Five putative *EILs* and 102 putative *ERFs* were recently reported in the peach genome [52]. Based on our previously reported database of the peach transcriptome [53], the expression levels of these *EIL* and *ERF* genes were estimated using the FPKM value (fragments per kilobase of transcript per million mapped reads) in different tissues, including green- and red-colored young leaves, white and red flowers, and yellow- and red-fleshed fruits. Among the five *EILs*, four members, *PpEIL1*, *PpEIL2*, *PpEIL3*, and *PpEIL5*, were highly expressed in fruit, with the FPKM value greater than 20.0, while one member *PpEIL4* showed no expression in all tested tissues (Table 1). *PpEIL1*, *PpEIL2*, and *PpEIL3* encode putative proteins of 601, 601, and 624 amino acids in length, respectively. By contrast, *PpEIL5* encodes a putative protein with only 101 amino acids in length due to a truncated EIN3 domain, suggesting the possibility of *PpEIL5* being a pseudogene. 

**Figure 1:**
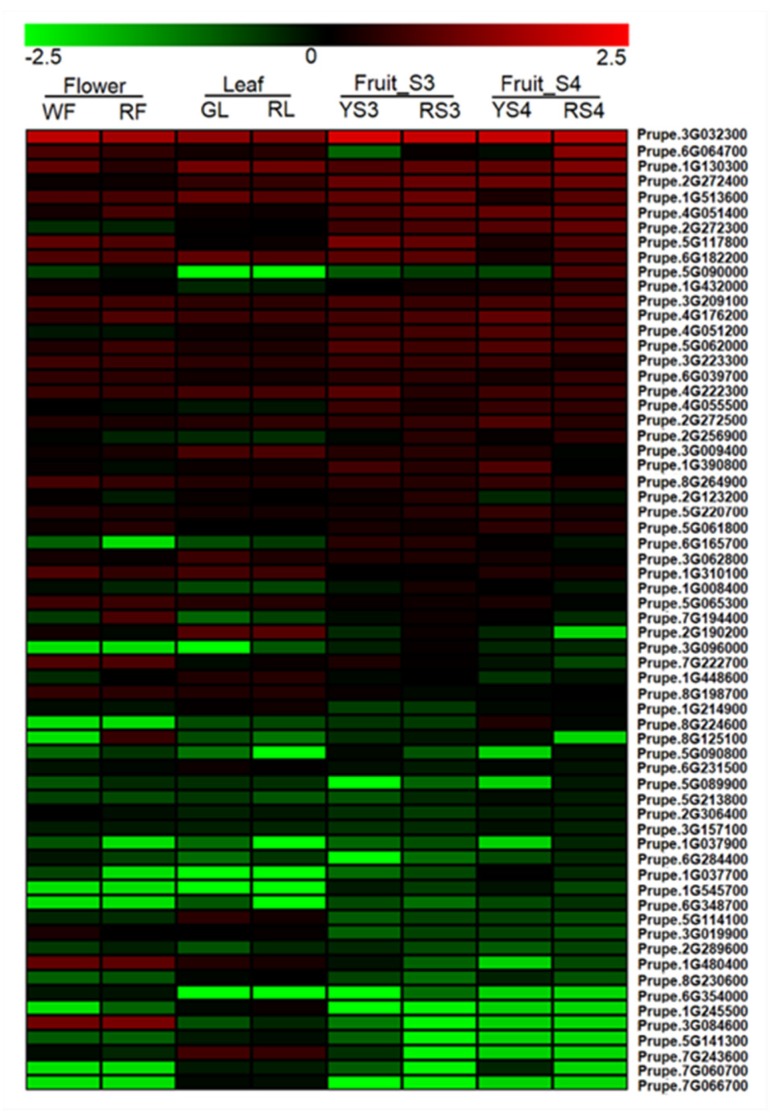
Heatmap of *ethylene response factor* (*ERF*) genes in the transcriptomes of different peach tissues. Red and green boxes indicate high- and low-expression levels, respectively. WF, white flower; RF, red flower; GL, green leaf; RL, red leaf; YS3, yellow-fleshed fruit at S3; RS3, red-fleshed fruit at S3; YS4, yellow-fleshed fruit at S4; RS4, red-fleshed fruit at S4. S3 and S4 represent the second exponential growth phase and ripening stage, respectively.

Out of the 102 *PpERFs*, 64 were expressed in at least one tissue, while 38 had no expression in all tested tissues. Subsequently, a heatmap was constructed to estimate the expression profile of these 64 expressed *PpERFs* based on the FPKM values (Figure 1). As a result, 12 *PpERFs* were found to be highly expressed in fruit, with FPKM > 10 in both yellow- and red-fleshed fruits at the ripening stage (S4), thus these were selected for further study. 

**Figure 2:**
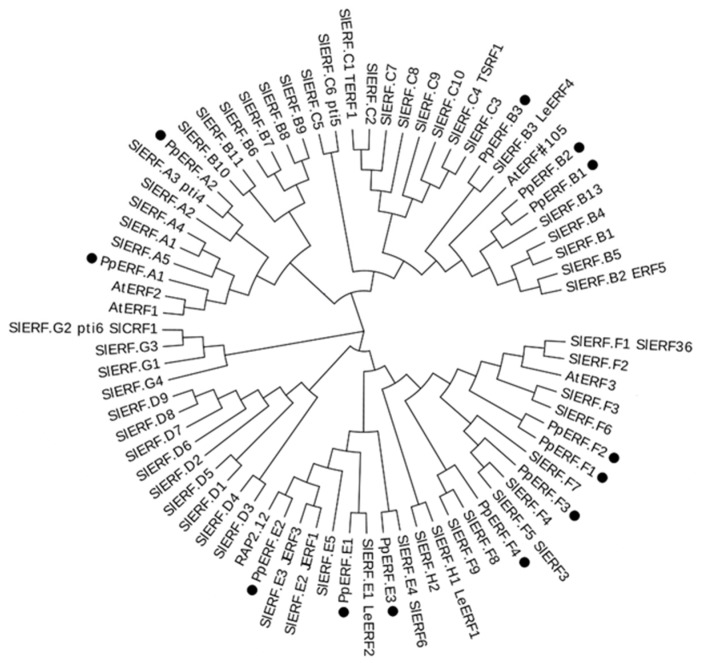
Phylogenetic tree derived from amino acid sequences of ERFs in peach, tomato, and *Arabidopsis*. The peach ERFs are highlighted with solid black circles. Full amino acid sequences of ERFs were aligned using Muscle software with default parameters, and a phylogenetic tree was constructed with MEGA software (version X) using the maximum likelihood method. All positions with less than 90% site coverage were eliminated, and ambiguous bases were allowed at any position (partial deletion option). The bootstrap consensus tree was inferred from 1000 replicates. All the tomato and *Arabidopsis* protein sequences were retrieved from Sol Genomics Network and Tair, respectively.

Phylogenetic analysis revealed that the 12 *PpERFs* were classified into four subgroups, A, B, E, and F (Figure 2). 

**Figure 3:**
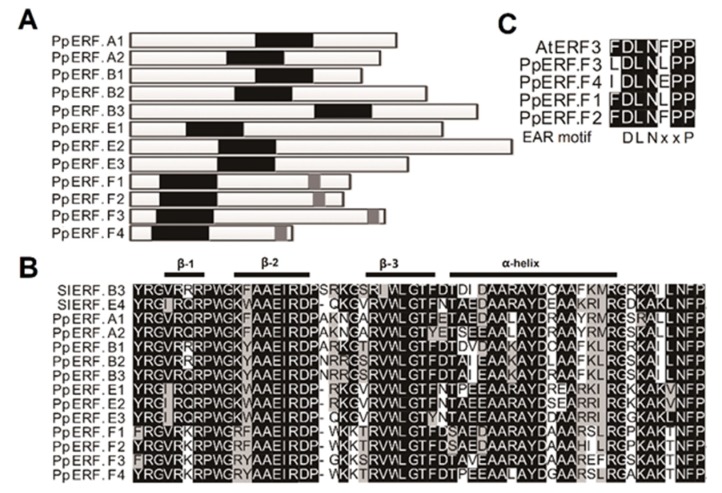
Diagram and alignment of ERF transcription factors (TFs). (**A**) structural diagram of the 12 *PpERFs* highly expressed in peach mesocarp. Black background boxes represent the AP2/ERF domain. The grey boxes stand for the EAR-repressing motif. (**B**,**C**) alignments of the EAR motif and AP2/ERF domain of the ERFs in peach and other species, respectively.

According to the nomenclature for the *ERF* family in tomato, these 12 *PpERFs* were designated as follows: *PpERF.A1* (Prupe.5G061800) and *PpERF.A2* (Prupe.2G272300) in subgroup A; *PpERF.B1* (Prupe.2G272500), *PpERF.B2* (Prupe.2G272400), and *PpERF.B3* (Prupe.5G062000) in subgroup B; *PpERF.E1* (Prupe.8G264900), *PpERF.E2* (Prupe.3G032300), and *PpERF.E3* (Prupe.1G130300) in subgroup E; and *PpERF.F1* (Prupe.3G209100), *PpERF.F2* (Prupe.4G222300), *PpERF.F3* (Prupe.4G051400), and *PpERF.F4* (Prupe.4G051200) in subgroup F. The deduced amino acid sequences of the 12 *PpERFs* all contained a conserved AP2/ERF domain with variable location sites (Figure 3A). The AP2/ERF domain consisted of three *β*-sheets and one *α*-helix (Figure 3B). In addition to the AP2/ERF domain, the EAR motif (DLNxxP), a repression domain, was also identified in subgroup F *PpERFs*, *PpERF.F1*, to *PpERF.F4* (Figure 3A,C). This suggested that the subgroup F *PpERFs* could have a negative regulatory activity.

### 2.2. Expression Profile of EIL and ERF Genes in Peach Fruit

**Figure 4:**
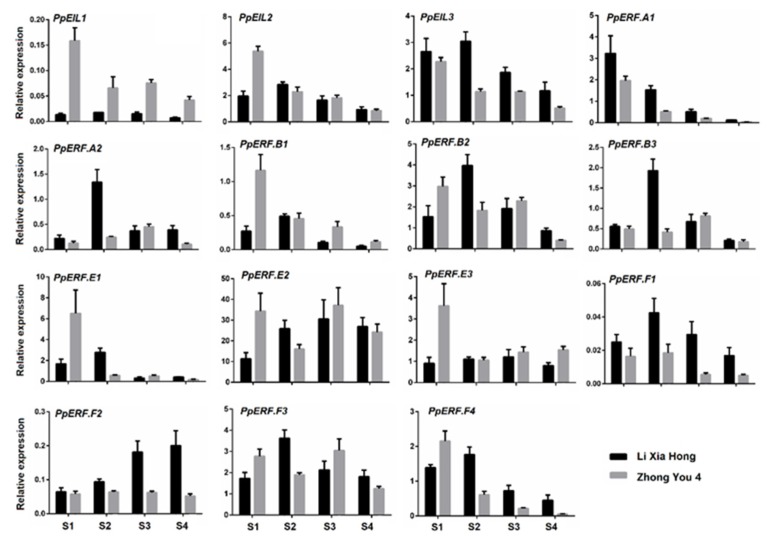
Expression profiles of the *PpEILs* and *PpERFs* in fruits of ‘Zhong You 4′ and its early ripening bud sport ‘Li Xia Hong’ at different developmental stages. The error bars show standard error (SE) of three biological replicates.

qRT-PCR was conducted to investigate the expression profile of *EIL* and *ERF* genes in fruits of cv. ‘Zhong You 4′ and its early ripening bud sport ‘Li Xia Hong’ at different developmental stages, including S1 (fruitlet), S2 (the first exponential growth phase), S3 (the second exponential growth stage), and S4 (the ripening stage). For the *PpEIL* genes, *PpEIL1* had extremely lower levels of expression compared with *PpEIL2* and *PpEIL3.* Both *PpEIL2 and PpEIL3* were constitutively expressed during fruit development and ripening, with *PpEIL3* showing consistently lower levels of expression in ‘Zhong You 4′ than in ‘Li Xia Hong’ (Figure 4).

A wide diversity of expression profiles were observed for the 12 *PpERFs* (Figure 4). Several *PpERFs* showed a decreasing trend in expression during fruit ripening. For example, the transcript abundance of three *PpERFs, PpERF.A1*, *PpERF.B1*, and *PpERF.E1*, was extremely low at the S4 stage of both ‘Zhong You 4′ and ‘Li Xia Hong’. By contrary, *PpERF.F2* showed an increasing trend in expression throughout the development of fruits in ‘Li Xia Hong’, with a peak at the fruit ripening stage. In addition, the expression levels of *PpERF.E2* remained consistently high throughout fruit development and were notably higher than those of other *PpERFs*.

### 2.3. Roles of PpEILs and PpERFs in Transcriptional Regulation of Ethylene Biosynthesis Genes

**Figure 5:**
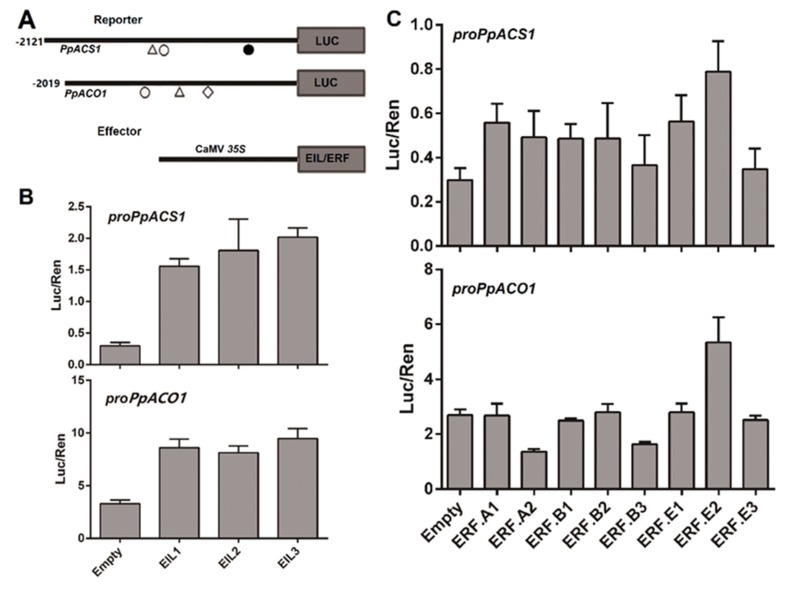
Functional analysis of the promoter sequences of *PpACS1* and *PpACO1*. (**A**) Construction of the effector and reporter cassettes. *Cis*-elements were indicated by triangle (DRE motif), black circle (PERE motif), white circle (PERE-like motif), and diamond (GCC-box). (**B**,**C**) Analysis of the interaction of PpEILs and activator-type PpERFs with the promoters of *PpACS1* and *PpACO1* using the dual luciferase assay in young leaves of 3- to 4-week-old *Nicotiana benthamiana* seedlings. The error bars represent SE of four biological replicates.

During the ripening process of fruits, *EILs* and *ERFs* participate in the fruit ripening process by regulating the expression of ethylene biosynthetic genes, *ACS* and *ACO* [7,18,26]. In peach, *PpACS1* and *PpACO1* have been proven to be the rate-limiting genes for system 2 ethylene biosynthesis [46,54]. To clarify the role of *PpEILs* or *PpERFs* in transcriptional regulation of *PpACS1* and *PpACO1*, *cis*-regulatory elements were screened in their promoter regions approximately 2-kb upstream of the start codon using the PLACE program (Figure 5A). Two putative PERE-like motifs located 453 and 1291 bp upstream of the start codon were identified for *PpACS1*, while only one putative PERE-like motif 1347 bp upstream of the start codon was found for *PpACO1*. Similarly, only one DRE box was found in the promoter of *PpACS1*, while one DRE and one GCC box were detected in the promoter of *PpACO1*. These results suggested potential roles of *PpEILs* and *PpERFs* in transcriptional regulation of *PpACS1* and *PpACO1*.

To validate whether *PpEILs* and *PpERFs* had a direct role in the transcriptional activation of *PpACS1* and *PpACO1* promoters, a dual luciferase assay was carried out. For the *PpACS1* promoter, Luciferase (Luc)/Renilla (Ren) values of *PpEIL1*, *PpEIL2*, and *PpEIL3* were 5.2-, 6.0-, and 6.7-fold higher, respectively, than that of the empty vector control (Figure 5B). For the *PpACO1* promoter, the Luc/Ren values of *PpEIL1*, *PpEIL2*, and *PpEIL3* were 2.6-, 2.5-, and 2.9-fold higher, respectively, than that of the empty vector control. These results indicated that *PpEIL1*, *PpEIL2*, and *PpEIL3* were able to activate the promoters of ethylene biosynthetic genes. Subsequently, the promoter activation activity was tested for eight activator-type *PpERFs* in subgroups A, B, and E (Figure 5C). Among the eight *PpERF* genes, *PpERF.E2* showed the highest ability to activate the promoters of both *PpACS1* and *PpACO1*, with a 2.6- and 2.0-fold higher Luc/Ren value, respectively, than the empty vector control. Moreover, *PpERF.E2* was phylogenetically related to tomato ripening-associated *ERFs*, such as *SlERF.E1*, *SlERF.E2*, and *SlERF6*/*SlERF.E4* (Figure 2), with 47.7%, 58.1%, and 37.8% amino acid sequence identities, respectively. Thus, *PpERF.E2* was deemed a candidate involved in ethylene synthesis in peach.

**Figure 6:**
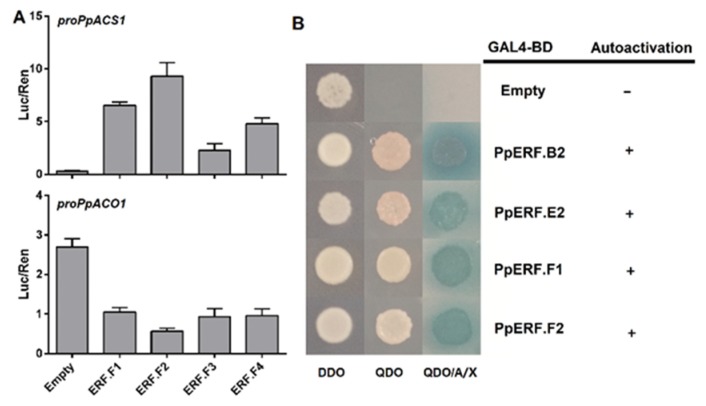
Functional analysis of group F ERFs. (**A**) Assay of the activation of group *F ERFs* on the promoter of *PpACS1* and *PpACO1* using the dual luciferase assay. Empty vector was used as the control. The error bars show SE of four biological replicates. (**B**) Auto-activation activity test of PpERFs in yeast. The yeast was grown on three medium Minimal Media Double Dropouts (DDO), Minimal Media Quadruple Dropouts (QDO), and QDO with Aureobasidin A and X-a-Gal (QDO/A/X), corresponding to SD-Trp, SD-Trp-His-Ade, and SD-Trp-His-Ade+ABA+X-α-Gal, respectively.

Finally, the promoter repressive activity was tested for four repressor-type *PpERFs* in subgroup F, including *PpERF.F1*, *PpERF.F2*, *PpERF.F3*, and *PpERF.F4*, which all harbored the EAR motif (Figure 3A). Among the four repressor-type *PpERF* genes, *PpERF.F2* showed the strongest repressive activity on the *PpACO1* promoter, with an approximately 5-fold lower Luc/Ren value than the empty vector control (Figure 6A). Surprisingly, all the subgroup F *PpERFs* showed high levels of activation activity instead of repressive activity on the *PpACS1* promoter. Among all the tested *PpERFs*, *PpERF.F2* showed the strongest ability to activate the *PpACS1* promoter, with a 30.0-fold higher Luc/Ren value than the empty vector control. Altogether, these results suggested that subgroup F *ERFs* in peach exhibit a potential dual function and could repress or activate gene transcription depending on the promoter in which they act.

### 2.4. Analysis of Transcriptional Activation Ability of PpERF Proteins in Yeast

The N- and C-terminal regions of ERF TFs contain transcriptional activation or repression domains [22]. To investigate whether subgroup F *PpERFs* also contain an activation domain, in addition to the EAR repression domain, full-length coding sequences of *PpERF.F1* and *PpERF.F2* were individually inserted into the pGBKT7 vector, and then transformed into yeast strain Y2Hgold to test their auto-activation activity in yeast cells. Activator-type ERFs, *PpERF.B2* and *PpERF.E2*, were used as positive controls. Yeast cells carrying either positive control genes or subgroup F *PpERF* genes were able to grow on the SD-Trp-His-Ade+ABA medium, and their color turned into blue when grown on media containing X-α-Gal (Figure 6B). This result suggested the presence of an activation domain, besides the EAR repressive domain, in proteins encoded by he *PpERF.F1* and *PpERF.F2* genes, which endowed them with potential dual functions: Acting as both an activator and repressor in peach.

### 2.5. Identification of Cis-Elements in the PpACS1 Promoter That Interact with EIL and ERF TFs

**Figure 7:**
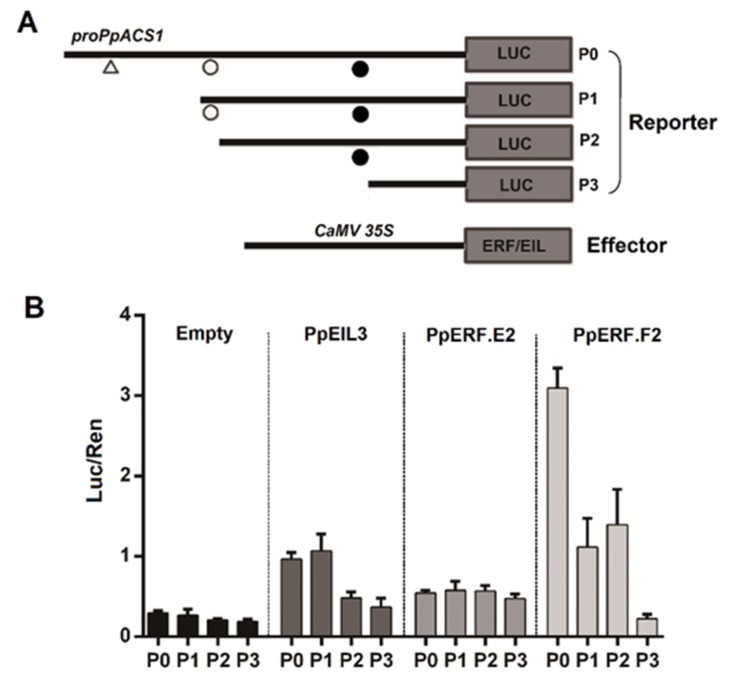
Promoter deletion analysis of *PpACS1*. (**A**) Four promoter deletion constructs used for the construction of reporter cassette and *ERF/EIL* genes related to the effector cassette. The predicted DRE, PERE-like, and PER motifs are indicated by a triangle, white circle, and black circle, respectively. (**B**) Assay of the activation effect of PpEIL3, PpERF.E2, and PpERF.F2 on four different length sequences of *PpACS1* using the dual luciferase assay. The error bars show SE of four biological replicates.

To identify *cis*-elements interacting with PpEILs, PpERF.E2 and subgroup F ERFs, four different promoter constructs of *PpACS1*, ranging from 0.4 to 2.1 Kb upstream of the start codon, were selected to conduct the dual luciferase assay (Figure 7A). PpEIL3 showed an almost equal ability to activate either P0 or P1 promoters but with a dramatically reduced ability to activate both P2 and P3 deletion constructs (Figure 7B). This indicated that PpEIL3 interacted with the PERE-like motif (ATTCAAA). Similarly, PpERF.F2 showed the strongest ability to activate the P0 promoter but with a dramatically reduced ability to activate P1 and P2 deletion constructs that both contained a deletion of the DRE motif (GTCGG) as compared to the P0 promoter. Therefore, PpERF.F2 is likely to interact with the DRE motif. However, PpERF.E2 showed the similar ability to activate all four promoters, suggesting that PpERF.E2 probably activated the *PpACS1* promoter in an indirect way. Taken together, these results suggested that *PpEILs* and *PpERFs* could regulate the transcription of *PpACS1* through either direct or indirect interaction with various *cis*-elements in the promoter region.

## 3. Discussion

The fruit ripening process in climacteric fruits is tightly associated with ethylene biosynthesis, which is mediated by the auto-inhibitory system 1 in unripe fruit and autocatalytic system 2 in ripening fruit [6,7]. Although a positive feedback loop between ethylene signaling and biosynthesis is known to control the autocatalytic system 2, its associated TFs and pathway are yet to be determined. A recent study showed that eudicots with or without recent whole-genome duplication (WGD) utilize the MADS- or NAC-type positive feedback loops, respectively, while monocots, such as banana, uses both MADS-box and NAC TFs to form a dual-loop system [55]. This suggests that *MADS-box* and *NAC* genes are involved in ethylene biosynthesis, acting as regulators that induce transcription of ethylene biosynthesis genes *ACS* and *ACO*. An MADS-type positive feedback loop seems to be true in tomato as the *MADS-box* gene *RIN* is weakly expressed in young fruit but highly expressed in mature green and breaker stages [56]. In peach, *PpNAC1* is reported to be involved in an NAC-type postive feedback loop [55]. However, *PpNAC1* shows low levels of expression in early stages of fruit development, but with a high level of expression in the second exponential growth stage (S3) before the initial activation of ethylene biosynthesis [53]. Thus, it is more likely that other regulatory gene(s) instead of *PpNAC1* may play critical roles in ethylene biosynthesis despite the transcriptional regulation of *ACS* and *ACO* by *PpNAC1* [55].

Besides *MADS-box* and *NAC* genes, other *TFs* at the late step of ethylene signal transduction, such as *EIN3/EIL* and *ERF*, are also known to participate in ethylene synthesis [17,18]. EIN3/EIL proteins are post-translationally regulated in response to the ethylene signal in *Arabidopsis* [57]. Several *EILs* that are constitutively expressed throughout fruit development have been reported in climacteric fruits, such as tomato and kiwifruit, although their expression levels are relatively lower during the ripening stage than in the early stages of fruit development [17,18]. A similar phenomena was also observed for peach *EILs* in this study. All three *PpEILs* can activate the transcription of both *PpACS1* and *PpACO1*, which is consistent with the previous finding in tomato and kiwifruit [7,18]. In *Arabidopsis*, ubiquitin or proteasome-dependent proteolysis mediates the post-translational regulation of EIN3 by two F-box proteins, EBF1 and EBF2 [58,59]. Given the fact that ethylene remains at a basal level in climacteric fruits at the juvenile stage, it is reasonable to speculate that the expression of *EILs* in climacteric fruits, such as peach, is also regulated at the post-translational level. Moreover, *EIN3/EIL* are known to activate ethylene response genes, such as *AtERF1*, *OsEBP-89*, and *NtTERF1*, through binding to the PERE or PERE-like *cis*-elements in their promoter regions [10,12,13]. Interestingly, PpEIL3 interacts with the PERE-like motif (ATTCAAA) found in the promoter of *TERF1*, which is regulated by EILs in tobacco, rather than the PERE motif (TTCAAAT) found in the promoter of *Os-EBP89* encoding an ethylene responsive element binding protein in rice [13]. Thus, it seems that the divergence of EIL-binding motifs has occurred during the process of speciation in plants. In addition, all three *EIL* genes can activate the promoters of *PpACS1* and *PpACO1*, suggesting a redundant role of the *EIL* gene family in ethylene biosynthesis.

The ERF family is divided into two subfamilies, CBF/DREB and ERF. Subgroups B and E of the ERF subfamily have been reported to participate in the fruit ripening process [22,60]. In tomato, a subgroup B member *Sl-ERF.B3* and a subgroup E member *LeERF2/TERF2* positively or negatively regulate the expression of both *ACS* and *ACO* genes, respectively [38,61], suggesting that subgroups B and E of the ERF subfamily may be involved in ethylene biosynthesis. Here, three subgroup E members, *PpERF.E1—PpERF.E3*, were highly expressed in fruit, but only *PpERF.E2* had ability to activate *PpACS1* and *PpACO1*. This suggests that *PpERF.E2* participates in ethylene biosynthesis. By contrast, three subgroup B members, *PpERF.B1* to *PpERF.B3*, which were highly expressed in fruit, were all unable to activate either *PpACS1* or *PpACO1* (Figure 5C). Previous studies show that *ERF* genes can form a hierarchical transcriptional cascade to regulate the ripening process in tomato [23] and apple [39]. Thus, it is worthy of further study to ascertain whether subgroup B members of the ERF subfamily can act as upstream regulators of *PpERF.E2*.

The F subgroup ERF TFs harboring the EAR motif (LxLxL or DLNxxP) in the C-terminus function as repressors to negatively regulate photosynthesis, stress response, cell wall metabolism, and fruit ripening [18,26,61]. Overexpression of the F subgroup *ERF TFs*, such as *ERF3* in cotton [35] and *MaERF11* in banana [62], results in downregualtion of *ACO* and *ACS*. Interestingly, our results showed that all the four F subgroup *ERF TFs* harboring the EAR motif, *PpERF.F1—PpERF.F4*, could play a dual function by activating *PpACS1* and repressing *PpACO1*. ERF TFs usually regulate fruit ripening by directly binding to the GCC-box or DRE motif of their downstream genes [38,39], and previously reported EAR-motif-containing ERF TFs are found to bind to the GCC box but not the DRE motif [42,62]. Nevertheless, one DRE *cis*-element instead of the GCC box in the promoter region of *PpACS1* is most likely interact with PpERF.F2 in this study, indicating the possibility that EAR-motif-containing ERF repressors directly activate *PpACS1* in peach. However, it cannot be ruled out that the peach ERF repressors indirectly activate *PpACS1* through interacting with other DRE-motif-containing regulators involved in fruit ripening.

## 4. Methods

### 4.1. Plant Material

Peach variety ‘Zhong You 4′ and its early ripening bud sport ‘Li Xia Hong’ are maintained in Institute of Horticulture, Anhui Academy of Agricultural Sciences, Hefei 230031, China. For ‘Zhong You 4′, fruit samples were collected at 15, 31, 47, and 63 days after flower bloom (DAFB), corresponding to S1 (fruitlet), S2 (the first exponential growth phase), S3 (the second exponential growth stage), and S4 (the ripening stage), respectively. For ‘Li Xia Hong’, fruit samples were also collected at 15 (S1), 31 (S2), 55 (S3), and 79 (S4) DAFB. Fruit samples were peeled, cored, and cut into small pieces; frozen immediately in liquid nitrogen; and then stored at -75°C until use. Each cultivar at each stage had three biological replicates, with each containing at least five fruits.

### 4.2. Heatmap Analysis of the ERF Gene Family

RNA-seq data were retrieved and analyzed according to our previous report [53]. Gene expression levels in leaves, flowers, and fruits were quantified using FPKM values (fragments per kilo bases per million reads). The heatmap was created using MeV (Multiple Experiment Viewer) 4.90 software [63].

### 4.3. RNA Extraction and Quantitative Real-Time PCR (qRT-PCR)

Approximately 100-mg fruit samples were ground into powder in liquid nitrogen and then subjected to total RNA extraction using the Total RNA Rapid Extraction Kit (Zomanbio, Beijing 100085, China) according to the manufacturer’s instructions. RNA extracts were treated with DNase I and then converted to cDNA using the PrimeScript^TM^ RT reagent Kit with gDNA Eraser (Takara, Dalian 116000, China). qRT-PCR was conducted using TB GREEN (Takara Bio, Inc.), following the manufacturer’s instructions on an ABI StepOne Plus real-time PCR system (Applied Biosystems, Foster City, CA, USA). The amplification program was as follows: One cycle of 30 s at 95 °C, followed by 40 cycles of 5 s at 95 °C and 34 s at 60 °C. Translation elongation factor *2* (*TEF2*) was used as a constitutive control according to the previous report [64]. All analyses were repeated three times using biological replicates. Primer sequences used for qRT-PCR are listed in Table S1.

### 4.4. Dual Luciferase Reporter Assay

The dual luciferase reporter assay was conducted as previous reports [65,66]. Briefly, the 2.1- and 2.0-kb regions upstream of the start codon in *PpACS1* and *PpACO1*, respectively, were amplified and inserted into the reporter vector pGreenII 0800-LUC+, whereas the effector vectors were constructed by insertion of the full-length coding sequences of *EIL* and *ERF* genes into the binary expression vector pSAK277 under the CaMV 35S promoter. Four *PpACS1* promoter deletion constructs, P0, P1, P2, and P3, containing 2.12-, 1.31-, 1.28-, and 0.45-kb DNA fragments upstream of the start codon, respectively, were selected to conduct the dual luciferase assay.

Both the reporter and effector constructs were individually transformed into *Agrobacterium* strain GV3101 harboring the pSoup helper plasmid. After incubation at 28 °C for 2 days, a 10-µL loop of confluent bacteria was re-suspended in 10 mL of infiltration buffer containing 10 mM MgCl_2_, 200 μM acetosyringone, and 10 mM 2-(*N*-morpholine)-ethanesulfonic acid (pH = 5.7), and incubated at room temperature without shaking for 2 h. *Agrobacterium* cultures containing the reporter cassette or the effector construct were mixed at a ratio of 1:9. The mixed cultures of bacteria were injected into young leaves of *Nicotiana benthamiana* seedlings by syringes without a needle. The seedlings were cultured in a greenhouse at 25 °C with a 16/8 h light/darkness cycle for 3 days. Leaf disks (2 cm in diameter) adjacent to the infiltration site were punched to measure the Luc and Ren luciferase activity using the Dual-Glo^®^ Luciferase Assay System (Promega, Madison, WI 53711, USA) on an Infinite M200 luminometer (Tecan, Mannerdorf, Switzerland).

### 4.5. Measurement of Transcriptional Activation Activity of ERFs in Yeast

The whole coding regions of *PpERF.B2*, *PpERF.E2*, *PpERF.F1*, and *PpERF.F2* were cloned, inserted into the pGBKT7 vector, and individually transformed into the yeast strain ‘Y2Hgold’ according to the instruction of the Frozen-EZ Yeast Transformation II^TM^ (ZYMO RESEARCH CORP, Irvine, CA 92614, USA). Positive clones were grown on the SD-Trp or SD-Trp-His-Ade+AbA medium to test the transcriptional activation activity. Photographs were taken 3 days after incubation on medium.

## 5. Conclusions

In total, 3 *PpEIL* genes and 12 *PpERF* genes showing relatively high expression in fruit were investigated for their relationship with ethylene biosynthesis in peach. All three EILs were able to activate the promoters of ethylene biosynthesis genes *PpACS1* and *PpACO1*, suggesting that they are potentially involved in ethylene biosynthesis. Of the 12 *PpERFs*, *PpERF.E2* was deemed a candidate involved in ethylene biosynthesis, as it showed a high expression as well as an ability to activate the transcription of *PpACS1* and *PpACO1*. Interestingly, four subgroup F PpERFs harboring the EAR repressive motif are not only able to repress the *PpACO1* promoter but can also activate the *PpACS1* promoter. Thus, *PpERFs PpERFs* could function as an activator or repressor of ethylene biosynthesis genes in peach. Our study provides an insight into the roles of *EILs* and *ERFs* in the fruit ripening of peach.

## Figures and Tables

**Table 1:** The Fragments Per Kilobase per Million (FPKM) value for the *PpEIL* genes in the leaf, flower, and fruit.

Gene	Accession No.	Flower		Leaf		Fruit			
		WF	RF	GL	RL	YS3	RS3	YS4	RS4
*PpEIL1*	Prupe.6G018200	13.2	13.0	13.5	15.6	12.5	15.6	15.3	21.6
*PpEIL2*	Prupe.2G058400	78.8	77.7	130.8	146.3	198.6	171.7	167.9	225.2
*PpEIL3*	Prupe.2G058500	42.9	35.8	68.9	68.9	61.5	44.0	46.8	65.5
*PpEIL4*	Prupe.6G181600	0.0	0.0	0.0	0.0	0.0	0.0	0.0	0.0
*PpEIL5*	Prupe.2G070300	14.8	13.4	19.2	23.1	22.2	22.6	8.1	30.6

WF, white flower; RF, red flower; GL, green leaf; RL, red leaf; YS3, yellow-fleshed fruit at S3; RS3, red-fleshed fruit at S3; YS4, yellow-fleshed fruit at S4; RS4, red-fleshed fruit at S4. S3 and S4 represent the second exponential growth phase and ripening stage, respectively.

## Supplementary Materials

The following are available online at https://www.mdpi.com/1422-0067/21/8/2846/s1, Table S1. Sequences of primers used for cloning genes and vectors construction and qRT-PCR.
