# Supplementary material for: Identification of EIL and ERF Genes Related to Fruit Ripening in Peach
Source: Int J Mol Sci. 2020 Apr 19;21(8):2846. doi: 10.3390/ijms21082846 (PMC7216043; doi:10.3390/ijms21082846)
Supplement: Supplementary file 1 [file ijms-21-02846-s001.pdf]

Table S1. Sequences of primers used for cloning genes and vectors construction and qRT-PCR

Primer name	Sequence	Description
PpERF.A1OEF	ccggaattcATGTTCGGACAGAGTGGAACAGA	Primers used for inserting PpERF.A1 into vector pSAK277
PpERF.A1OER	gctctagaTCAGCTCACTAATAACTGCTCGC	
PpERF.A2OEF	ccggaattcATGATTATGAGTGATAATAATCAATCTCTGA	Primers used for inserting PpERF.A2 into vector pSAK277
PpERF.A2OER	gctctagaCTAGCTCACCAATAGTTGCTCGC	
PpERF.B1OEF	ccggaattcATGCATGCCAATCAACTCTCCT	Primers used for inserting PpERF.B1 into vector pSAK277
PpERF.B1OER	gctctagaTCAAGGCTTCAAGAGTTCGGTT	
PpERF.B2OEF	ccgctcgagATGGCAACAGAGCATGAAGAAAC	Primers used for inserting PpERF.B2 into vector pSAK277
PpERF.B2OER	gctctagaTTATGTAACCATAAGCTGAGGAAATCC	
PpERF.B3OEF	cccaagcttATGGCTTTGAGCTCAGACATGG	Primers used for inserting PpERF.B3 into vector pSAK277
PpERF.B3OER	gctctagaTCACACGACCATAAGTTGTGGAT	
PpERF.E1OEF	ccggaattcATGTGTGGCGGTGCTATTCTCT	Primers used for inserting PpERF.E1 into vector pSAK277
PpERF.E1OER	gctctagaTTACGGAGCAGAAACGCGGT	
PpERF.E2OEF	ccgctcgagATGTGTGGAGGTGCTATAATATCCG	Primers used for inserting PpERF.E2 into vector pSAK277
PpERF.E2OER	gctctagaTCAGAAACCTCCCCCAATCAG	
PpERF.E3OEF	ccggaattcATGTGTGGTGGCGCTATCATT	Primers used for inserting PpERF.E3 into vector pSAK277
PpERF.E3OER	gctctagaTTAATACAGCTGCTGACCCTGCT	
PpERF.F1OEF	ccggaattcATGCGGCGAGGAGGCAGA	Primers used for inserting PpERF.F1 into vector pSAK277
PpERF.F1OER	gctctagaTTAGAGGCACAAAGCGGTGG	
PpERF.F2OEF	ccggaattcATGAGGAGGGGCAGAGGAGC	Primers used for inserting PpERF.F2 into vector pSAK277
PpERF.F2OER	gctctagaTCAGAGGCATAGAGCCGTACATC	
PpERF.F3OEF	ccggaattcATGGCGCCGAGAGAGAAGAC	Primers used for inserting PpERF.F3 into vector pSAK277
PpERF.F3OER	gctctagaTCAAGCGAGCTCCGGAGGT	
PpERF.F4OEF	ccggaattcATGGCGACTGCTTCTTCATCTC	Primers used for inserting PpERF.F4 into vector pSAK277
PpERF.F4OER	gctctagaTCACAGCCACAAGGGTGGG	
PpEIL1OEF	cccaagcttATGGGTGACGTTGAAGAGGTTG	Primers used for inserting PpEIL1 into vector pSAK277
PpEIL1OER	ccgctcgagTTATGCCGCAAAGTATTCCATC	
PpEIL2OEF	ccggaattcATGGGGATCTTTGAAGAAATGG	Primers used for inserting PpEIL2 into vector pSAK277
PpEIL2OER	gctctagaTCAGATCCAAAATGCATCTTGC	
PpEIL3OEF	ccggaattcATGGGGATCTTTGAGGAAATGG	Primers used for inserting PpEIL3 into vector pSAK277
PpEIL3OER	gctctagaTTATGGGAACCAAAAGGATGCA	
proACS1P0F	cgcggatccTCCGTTCCTTGGCACTATGGT	Forward primer for proACS1P0-pGreenII0800LUC+
proACS1P1F	cgcggatccGAAGTAATCGGTGACATGTTAAATTC	Forward primer for proACS1P1-pGreenII0800LUC+
proACS1P2F	cgcggatccCTATACTAATATGGGCCAAACCAAT	Forward primer for proACS1P2-pGreenII0800LUC+
proACS1P3F	cgcggatccAATAGATATATCGTTAAATTTATTGTGTGAA	Forward primer for proACS1P3-pGreenII0800LUC+
proACS1R	GAGCCCATGGTCTTGGTTCCAAAG	Reverse primer for proACS1-pGreenII0800LUC+
ProACO1F	cgcggatccTTGGCTAAGTCACATCAAGGCA	Forward primer for proACO1-pGreenII0800LUC+
ProACO1R	GTTCTCCATGGCTCTCTCTCTCTTTGTG	Reverse primer for proACO1-pGreenII0800LUC+
PpERF.B2BDF	ggaattccatatgATGGCAACAGAGCATGAAGAAAC	Primers used for inserting PpERF.B2 into vector pGBKT7
PpERF.B2BDR	acgcgtcgacTTATGTAACCATAAGCTGAGGAAATCC	

PpERF.E2BDF	ggaattccatatgATGTGTGGAGGTGCTATAATATCCG	Primers used for inserting PpERF.E2 into vector pGBKT7
PpERF.E2BDR	acgcgtcgacTCAGAAACCTCCCCCAATCAG	
PpERF.F1BDF	ggaattccatatgATGCGGCGAGGAGGCAGA	Primers used for inserting PpERF.F1 into vector pGBKT7
PpERF.F1BDR	acgcgtcgacTTAGAGGCACAAAGCGGTGG	
PpERF.F2BDF	ggaattccatatgATGAGGAGGGGCAGAGGAGC	Primers used for inserting PpERF.F2 into vector pGBKT7
PpERF.F2BDR	acgcgtcgacTCAGAGGCATAGAGCCGTACATC	
PpEIL1rF	AGTGAGCGGTGCTTCTGATAAC	Primers used for qRT-PCR
PpEIL1rR	CGCCTCATACTTGGCTATGG	
PpEIL2rF	GCCTACTGGTAATGAGGAATGG	Primers used for qRT-PCR
PpEIL2rR	GCTTTATCACAGCCGTGAGAAC	
PpEIL3rF	GGTCCTCCTCCATACAAGAAGC	Primers used for qRT-PCR
PpEIL3rR	GCAGACATTTAGACTGACGAACAA	
PpERF.A1rF	CGTGCTTGACCGAAAACTGG	Primers used for qRT-PCR
PpERF.A1rR	GCGTCCCTTAGAACACCGTAG	
PpERF.A2rF	AACAGAACAGTGGGGAGATTTG	Primers used for qRT-PCR
PpERF.A2rR	AAGCTGTGATCGGGTAACCAG	
PpERF.B1rF	TGAACTCATTCGCCAGCATCT	Primers used for qRT-PCR
PpERF.B1rR	GAGGGAGCACTCTGGTTTCAC	
PpERF.B2rF	CAAAACCACCCAGACACTCCA	Primers used for qRT-PCR
PpERF.B2rR	TTCGACCACCACGTTGTTAGA	
PpERF.B3rF	CGAACGCATCAAGAACCATC	Primers used for qRT-PCR
PpERF.B3rR	TGGAGCAAAGCGAGTCAGAT	
PpERF.E1rF	TCCCAGCCCACTCACTCG	Primers used for qRT-PCR
PpERF.E1rR	CTGCCTGATGCCTCGGTAG	
PpERF.E2rF	TCTGATGTGGACGAAGATGATG	Primers used for qRT-PCR
PpERF.E2rR	TCAGCCTGCCCATTGAACT	
PpERF.E3rF	CTCCTTGGCTTAGACCACTCC	Primers used for qRT-PCR
PpERF.E3rR	GCACCTGGGCTCGTCTTCT	
PpFRF.F1rF	GATGTCGTTCAGGCAAGCAG	Primers used for qRT-PCR
PpFRF.F1rR	CAGTCGCTGTGGCAGTCATC	
PpFRF.F2rF	GTTTGGCTCGGCACCTTC	Primers used for qRT-PCR
PpFRF.F2rR	TGGTCGGTTGGGTTGTTGT	
PpFRF.F3rF	AATAGTAGCGGGAGCAACAATC	Primers used for qRT-PCR
PpFRF.F3rR	GGTACTAACACCCTGAGCGAGA	
PpFRF.F4rF	CACTACAGAGGCGTAAGGAAGAG	Primers used for qRT-PCR
PpFRF.F4rR	TCGAATGTACCCAACCAAACC	
